# Editorial: Molecular architects of the green world: genetic, epigenetic, and transcriptional regulation of plant metabolism and chemo-diversity

**DOI:** 10.3389/fpls.2026.1824488

**Published:** 2026-03-24

**Authors:** Bowei Chen, Liping Zhao, Shahid Ali, Shahbaz Chishti, Ryo Fujimoto, Min Wang, Fen Liu, Qingzhu Zhang

**Affiliations:** 1 Laboratory of Plant Resource Conservation and Utilization, School of Life Sciences, Jishou University, Jishou, China; 2 Institute of Carbon Neutrality, Key Laboratory of Sustainable Forest Ecosystem Management-Ministry of Education, School of Ecology, Northeast Forestry University, Harbin, China; 3 Heilongjiang Maoershan Forest Ecosystem National Observation and Research Station, School of Ecology, Northeast Forestry University, Harbin, China; 4 Guangxi Key Laboratory of Agro-Environment and Agro-Products Safety, College of Agriculture, Guangxi University, Nanning, China; 5 Graduate School of Agricultural Science, Kobe University, Kobe, Japan; 6 College of Agriculture and Forestry Science and Technology, Hebei North University, Zhangjiakou, China; 7 College of Pharmacy, Jishou University, Jishou, China

**Keywords:** plant metabolism, genetic codes, transcriptional regulation, epigenetic regulation, omics analysis, chemo-diversity

The plant kingdom works like a sophisticated chemical factory, where species ranging from dwarf herbs and shrubs to towering trees continuously synthesize a vast array of metabolites characterized by complex structures and diverse biological functions throughout their life (Weng et al., 2021). Traditionally, plant metabolism can be divided into primary and secondary metabolism, yet the boundary between these two has become increasingly difficult to distinguish (Palazon and Alcalde, 2025). These metabolites not only play a crucial role in plant growth and development but also serve as vital defense mechanisms enabling plants to cope with biotic and abiotic stresses and adapt to local climate change (Khan et al., 2025). The question now, however, is: How are this magical plant metabolites designed? In recent years, with the integration of multi-omics and molecular biology, scientists have been uncovering the blueprint of this metabolic biosynthesis at a high resolution, that is a “molecular architecture” governed by the genetic codes, transcriptional and epigenetic regulation. This editorial will focus on a series of recent studies that explore plant metabolic diversity with multi-omics methods, aiming to systematically elucidate the multi-layered complexity of its regulatory network and highlight tremendous potential for understanding plant chemical diversity.

## The genetic basis of plant metabolic diversity

The core backbone of plant metabolic pathways is composed of a series of biosynthetic enzymes. For example, anthocyanins and lignin, key contributor to flower coloration and secondary wall formation, are synthesized through the phenylpropanoid pathway, in which chalcone synthase (CHS) play central role (Deng and Lu, 2017). Meanwhile, terpenoid compounds, which play roles in antioxidant defense, signal transduction, and stomatal movement, are derived from the mevalonate pathway involving farnesyl diphosphate synthase (FDPS) (Cheng et al., 2025). The variation in copy numbers, sequence diversity, gene family expansion or contraction of these functional genes encoding key biosynthetic enzymes constitute the fundamental genetic basis for chemical diversity both between and within plant species.

In this editorial, Cao et al. revealed that the mild aroma of *Artemisia stolonifera* results from the up-regulation of mevalonate pathway (e.g., *AACT1–4* and *HMGR1-3*) and sesquiterpene synthase genes, leading to the accumulation of sesquiterpenoids. In contrast, the wild aroma of *A. argyi* is attributed to the methylerythritol phosphate pathway (e.g, *DXS2* and *DXR1-5*) and enhanced monoterpenoid and flavonoid biosynthesis, resulting in the enrichment of monoterpenoids and flavonoids. Wu et al. found that the higher oxidation resistance of white *Paeonia lactiflora* compared to other varieties is linked to its higher content of paeoniflorin, polyphenols and flavonoids. Similarly, Zhang et al. demonstrated that diverse sugarcane varieties adopted distinct adaptive molecular reprogramming strategies in response to ratooning decline. In addition, Liu et al. identified a locus, *UGT-Rd1*, that is significantly associated with the protopanaxadiol ginsenoside Rd content. Its genetic variants not only obviously influence the efficiency of Rd ginsenoside biosynthesis but also exhibit specific genetic effects. Collectively, these findings suggest that metabolic diversity among different varieties is largely shaped by the genetic architecture of their biosynthetic enzymes. However, the ‘chemical architecture’ that is ultimately constructed also relies on higher-level and more dynamic regulatory mechanisms.

## Transcriptional regulation of plant metabolic diversity

Over the past decades, numerous transcription factors (TFs), such as MYB, bHLH, and WD40 members, have been demonstrated to play vital roles in regulating metabolic pathways including phenylpropanoid metabolism, the mevalonate pathway, alkaloid biosynthesis, epidermal wax biosynthesis, and glucosinolate biosynthesis (Xu et al., 2015). Such transcriptional regulation often shows tissue specificity. For example, Zhang et al. discovered a molecular regulatory network directed by ARF, bHLH, GRAS, G2-like, and ARR-B TFs, which is significnatly associated with the spatially distribution patterns of auxin (IAA), gibberellin (GA), and cytokinin (CTK) in *Cinnamomum burmanni*. Furthermore, alternative splicing (AS) can also contribute to diversity in the protein structure and biological function of TFs. An et al. found that the alternative transcripts of functional genes in the phenylpropanoid metabolism pathway including *4CL*, *CHS*, *F3H*, *DFR*, *GT*, and *ANR*, as well as the regulatory factor *bHLH*, exhibit a significant positive correlation with flavonoid content in different tissues of *Rosa roxburghii*.

Transcriptional regulation of plant metabolic diversity also exhibits time-course characteristics. For example, Zhang et al. discovered that during the R3 stage of sugarcane ratoon decline, core genes such as galactinol synthase homologs, thiosulfate sulfurtransferase genes, and the E3 ubiquitin ligase gene may participate in mitigating the decline process through distinct molecular strategies involving the regulation of osmotic tolerance, oxidative defense, and protein homeostasis. This finding suggests that the reduction in sugarcane ratoon yield is closely related to a shift in root transcriptional regulation from growth maintenance toward senescence defense. In addition, Lei et al. highlighted that the arginine and phenylalanine content in *Amygdalus mongolica* kernels increased along with their development, closely linked to dynamic changes in their biosynthesis genes such as *glyA*, *SHMT*, *TYRAAT*, *YUCCA* and *SHMT*, and their transcriptional regulation.

Transcriptional regulation of plant metabolic diversity can be further affected by biotic and abiotic stresses. Bai et al. reported that under long-term salt stress, tobacco accumulates galactose, starch, and sucrose, and exhibits dynamic changes in the expression profiles of a series of functional genes, including sucrose-phosphate synthase (SPS), along with the activation of TFs associated with carbohydrate metabolism. Similarly, Wu et al. found that the biosynthesis of various acids and alcohols in *Paeonia lactiflora* root is likely influenced by interactions with the inter-root microbial communities such as *Acidobactiera* and *Proteobacteria*, which in turn affects plant antioxidant resistance.

## Epigenetic regulation of plant metabolic diversity

Epigenetics refers to reversible chemical modifications that alter gene expression without changing the DNA sequence, thereby leading to phenotypic variation or function changes. Recently, numerous studies have confirmed that epigenetic modifications, including DNA methylation, histone modifications, and non-coding RNAs, can serve as molecular switches in regulating the biosynthesis of plant metabolites (Cui et al., 2026). Furthermore, in the field of synthetic biology and molecular breeding, epigenetics also provides novel strategies for achieving stable and highly efficient expression of exogenous genes in transgenic plants. For example, Li et al. introduced synonymous mutations at the miRNA binding site of the walnut-derived growth-regulating gene *GRF4b*, therefore enabling it to escape from cleaving by endogenous miRNAs. This approach significantly enhanced the efficiency of genetic transformation in birch and effectively promoted callus growth and bud development. It suggests that systematically assessing the post-transcriptional epigenetic modification between trans-genes and endogenous non-coding RNAs in host plants is essential for achieving high expression levels in transgenic systems and ensuring the normal growth and development.

## Prospects: advancing multi-omics integration and medicinal plant research

A prominent trend in this topic is the extensive adoption of multi-omics methods, such as transcriptomics and metabolomics. With the continuous advancement and innovation in high-throughput sequencing and bioinformatics technologies, future investigations into plant metabolic diversity will increasingly integrate a wider range of omics technologies, such as genomics, proteomics, metagenomics, microbiomics, and pangenomics, and be combined with higher-resolution techniques such as single-cell sequencing (Hao et al., 2025). In addition, computational biology, systems biology, and artificial intelligence (AI) will play crucial roles in analyzing these large−scale datasets and building predictive regulatory network models (Holst et al., 2025). Another distinctive feature of this topic is that most studied plants belong to medicinal taxa, such as *Paeonia lactiflora*, *Amygdalus mongolica*, *Cinnamomum burmannii*, *Artemisia stolonifera*, *Panax ginseng*, and *Vanilla planifolia*. Research on the biosynthetic pathways of secondary metabolites, particularly the pharmacologically active components, in these medicinal plants holds substantial theoretical and practical significance. These studies will help clarify the origins of active ingredients, improve the quality of medicinal resources, and may ultimately achieve the efficient and sustainable production of bioactive compounds via synthetic biology strategies.

Unraveling how these “molecular architects of the green world” operate not only satisfies scientific curiosity about the mysteries of nature but also support sustainable agriculture in the face of growing climate challenges and food−security demands. It can pave the way for developing novel drugs and green chemicals, while contributing to plant−based carbon−neutral solutions. This journey from deciphering the codes of life to designing biological structures and functions is already underway, and the studies presented in this topic marks significant strides along this promising path.
